# Supplementary material for: Alfalfa Spring Black Stem and Leaf Spot Disease Caused by Phoma medicaginis: Epidemic Occurrence and Impacts
Source: Microorganisms. 2024 Jun 24;12(7):1279. doi: 10.3390/microorganisms12071279 (PMC11279198; doi:10.3390/microorganisms12071279)
Supplement: Supplementary file 1 [file microorganisms-12-01279-s001.zip › microorganisms-3004474-supplementary.pdf]

Figure 2. Methodology information

Plugs of hyphae (4 mm in diameter) of each of 64 isolates were transferred to sterile Petri dishes containing 15 mL oatmeal agar, potato carrot agar, potato dextrose agar, potato sucrose agar, malt extract agar, and czapek dox agar, and were subsequently incubated at 25°C to assess the effect of different substrates on the growth of *P. medicaginis* mycelium. Each isolate was replicated four times. There were 4 replicates in the experiment and all photos are for the same isolate and representative considering the variation across replicates.

Figure 3. Methodology information

Plugs of hyphae (4 mm in diameter) of each of 64 isolates were transferred to sterile Petri dishes containing 15 mL potato dextrose agar and were subsequently incubated at 5°C, 10°C, 15°C, 20°C, 25°C and 30°C to assess the effect of different temperatures on the growth of P. medicaginis mycelium. There were 4 replicates of each isolate-temperature combination and 1536 plates in total. Six incubators were used, each set to one of the six culture temperatures (256 plates per incubator). All photos are for the same isolate and representative, considering the variation across replicates.
